# The contribution of lysophosphatidic acid receptors in the response of human lower esophageal sphincter under the electrical field stimulation

**DOI:** 10.1186/s12876-023-02738-y

**Published:** 2023-05-18

**Authors:** Yong Feng, Wei Wei, Liang Chen, Jun-Feng Liu

**Affiliations:** 1 Department of Thoracic Surgery, Fourth Hospital of Hebei Medical University, 12 Jiankang Road, Shijiazhuang, 050011 Hebei China; 2 Outpatient Department, Fourth Hospital of Hebei Medical University, 12 Jiankang Road, Shijiazhuang, 050011 Hebei China; 3 Department of Thoracic Surgery, Hebei Chest Hospital, Shijiazhuang, 050011 Hebei China

**Keywords:** Clasp fibers, Electrical field stimulation, Lower esophageal sphincter, Lysophosphatidic acid receptor, Sling fibers

## Abstract

**Background:**

This study aims to identify the impact on the reaction while the clasp and sling fibers of the human lower esophageal sphincter are under the electrical field stimulation, by adding lysophosphatidic acid receptor subtypes antagonist.

**Methods:**

Between March 2018 to December 2018, muscle strips were isolated from 28 patients who underwent esophagectomy for mid-third esophageal carcinomas. Muscle tension measurement technique in vitro and electrical field stimulation were used to examine the effects of selective lysophosphatidic acid receptor antagonist on the clasp and sling fibers of human lower esophageal sphincter.

**Results:**

The optimal frequency of frequency-dependent relaxation in clasp fibers and contraction in sling fibers induced by electrical field stimulation is 64 Hz and 128 Hz respectively. The selective lysophosphatidic acid 1 and 3 receptor antagonist produced no significant difference in the frequency-dependent relaxation in clasp fibers and contraction in sling fibers induced by the electrical field stimulation (*P* > 0.05).

**Conclusion:**

The electrical field stimulation induced a frequency-dependent relaxation in clasp fibers and contraction in sling fibers. The lysophosphatidic acid 1 and 3 receptors are not involved in the response of clasp and sling fibers of the human lower esophageal sphincter induced by the electrical field stimulation.

## Introduction

Electrical field stimulation (EFS) can activate the ions in nerve fibers to move along the axons, form depolarization, and finally form nerve reflex [1]. The study of esophagus shows that EFS can simulate the neural reflex in human body, stimulate the enteric motor neurons (EMN) in the esophageal myometrium, and activate the neural conduction pathway. It was found that the nerve reflex induced by EFS on the esophageal smooth muscle in vitro innervated by the central nervous system can maintain the peristalsis of the esophagus [2], which indicates that EFS activates the nerve signal transduction pathway through the stimulation of EMN. Therefore, EFS is widely used in the study of regulatory mechanism of the lower esophageal sphincter (LES), a special muscle located at the esophagogastric junction. The human LES contains clasp and sling fibers [3].

Some studies have found that in the muscle wall layer of LES, the output nerve terminals of vagus nerve have synaptic connections with EMN, which together constitute the nerve conduction pathway regulating the movement of LES. It includes excitatory vagus nerve pathway and inhibitory vagus nerve pathway. The excitatory vagus nerve pathway comprises preganglionic cholinergic neurons and postganglionic cholinergic neurons. Activation of this pathway can cause postganglionic neurons to release neurotransmitters such as acetylcholine and substance P [4], which leads to LES contraction. The inhibitory vagus nerve pathway comprises preganglionic cholinergic neurons and postganglionic nonadrenergic noncholinergic (NANC) [5]. Activation of this pathway can cause postganglionic neurons to release neurotransmitters such as nitric oxide (NO) and vasoactive intestinal peptide (VIP), which leads to relaxation of LES. After the activation of LES nerve conduction pathway, the excitatory or inhibitory neurotransmitters released by postganglionic neurons not only include these neurotransmitters [6], but their specific types are not clear. These neurotransmitters are only confirmed to exist, and play an essential role in the regulation of LES. Whether lysophosphatidic acid (LPA) is also involved in the regulation of the neural pathway of LES has not been clearly reported.

In previous studies, we found that LPA receptors exist in clasp fiber and sling fiber of human LES, and the smooth muscle type LPA receptors were LPA 1R, LPA2R and LPA3R, respectively [7]. LPA induces contraction effect of clasp and sling fibers through LPA receptor. In the present study, we aimed to detect the effects of selective LPA receptor antagonists on clasp and sling fibers of human LES induced by the EFS.

## Patients and methods

### Patients and patient tissue

This present study was conducted at Department of Thoracic Surgery of Fourth Hospital of Hebei Medical University between March 2018 and December 2018. Muscle strips were collected from 28 patients (18 males, 10 females; mean age: 59 ± 12.1 years) who underwent an esophagectomy for mid-third esophageal carcinomas. All patients did not have these symptoms such as acid regurgitation and heartburn before operation, and there was no medical history and medication history that might lead to esophageal motor disorders. Fresh esophagogastric junction specimens were collected in the operating room, and the sling and clasp muscle strips were separated and cut into 2 × 10 mm. The muscle strips were made in the same way described previously [8, 9].

### The most suitable initial length

Both ends of the muscle strips were fastened with silk, and placed in a 10 ml bath containing Krebs liquid, maintained a constant temperature of 37 ℃ and persisted through the gas containing 5% CO2 and 95% O2. The upper of muscles and JZ101 type muscle tension transducer (Xinhang Electric Apparatus, Gaobeidian, China) were fastened together, the lower end of the muscle strip needs to be fixed on an L-shaped fixing frame with the platinum electrode. The muscle strip is located between two parallel annular platinum electrodes, and the distance between the upper and lower ends of the muscle strip and the electrode ring should be more than 3 mm. The platinum electrode is connected to the physiological and pharmacological multipurpose instrument for EFS. Muscle tension was recorded using the software of MedLab 6.0 software (MedEase, Nanjing, China).

**Fig. 1:**
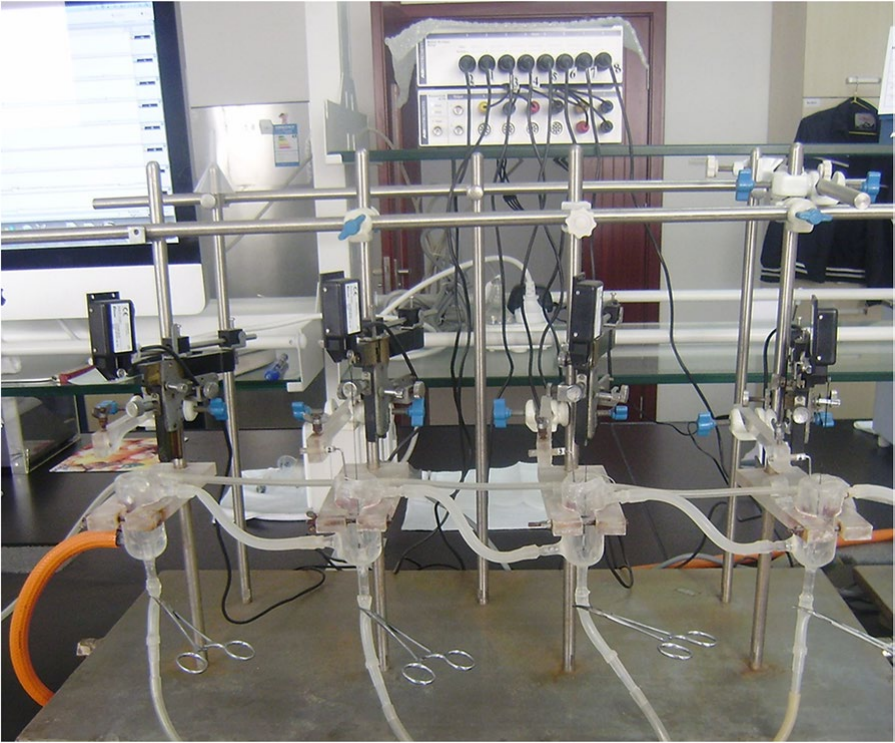
The organ bath of muscle strips

Each muscle strip was stretched slightly and rapidly until 200 mg of force was generated. This was taken as the initial length (L_0_). The muscle strips were then sequentially stretched to 200% of the L_0_ [10], at increments of 25% of the L_0_ each time [11]. This was taken as the most suitable initial length. Then the muscle strips were bathed in Krebs solution at 37℃ for 1 h continuously aerated with 95%O2 and 5%CO2 (Fig. 1).

### EFS and effect of selective LPA receptor antagonist on human LES

EFS stimulation parameters: monopulse square wave, wave width 5 ms, voltage 50 V, frequency 1 ~ 512 Hz, increasing in multiples [2]. The maximum effect after stimulation (Emax) was calculated. After the stimulation stopped and the muscle strips were restored to balance, the selective LPA1 receptor and LPA3 receptor antagonist ki16425(Cayman Chemical, Ann Arbor, MI, USA) (10^–5^ mol/L) were added into the bath. EFS was performed after the drug was added 20 min, and the response of clasp and sling fibers muscle strips by EFS before and after the drug was compared.

The EFS experiment requires that in the process of preparing clasp fiber and sling fiber muscle strips of human LES, 2 × 10 mm muscle strips must be strictly prepared. Because the distance between two parallel annular electrodes is 30 mm, its radius is 2 mm. This makes the muscle strip have no direct contact with the electrode ring when it reaches the most suitable initial length (200% of the initial length L0), and ensures that the distance between its upper and lower ends and the electrode ring is more than 3 mm. It avoids the contraction or relaxation reaction of muscle strips induced by direct electrical stimulation, and reduces the interference factors in the experiment.

### Statistical analysis

Statistical analysis was performed using the IBM SPSS version 19.0 (IBM Corp., Armonk, NY, USA) and GraphPad Prism version 5.0 software (GraphPad Software Inc., San Diego, CA, USA) [7]. Descriptive data were expressed in mean ± standard error (SEM). Two-way analysis of variance (ANOVA) was used to compare the two drug concentration–response curves. A *p* value of < 0.05 was considered statistically significant.

## Results

### Effect of EFS on the clasp and sling fibers

**Fig. 2:**
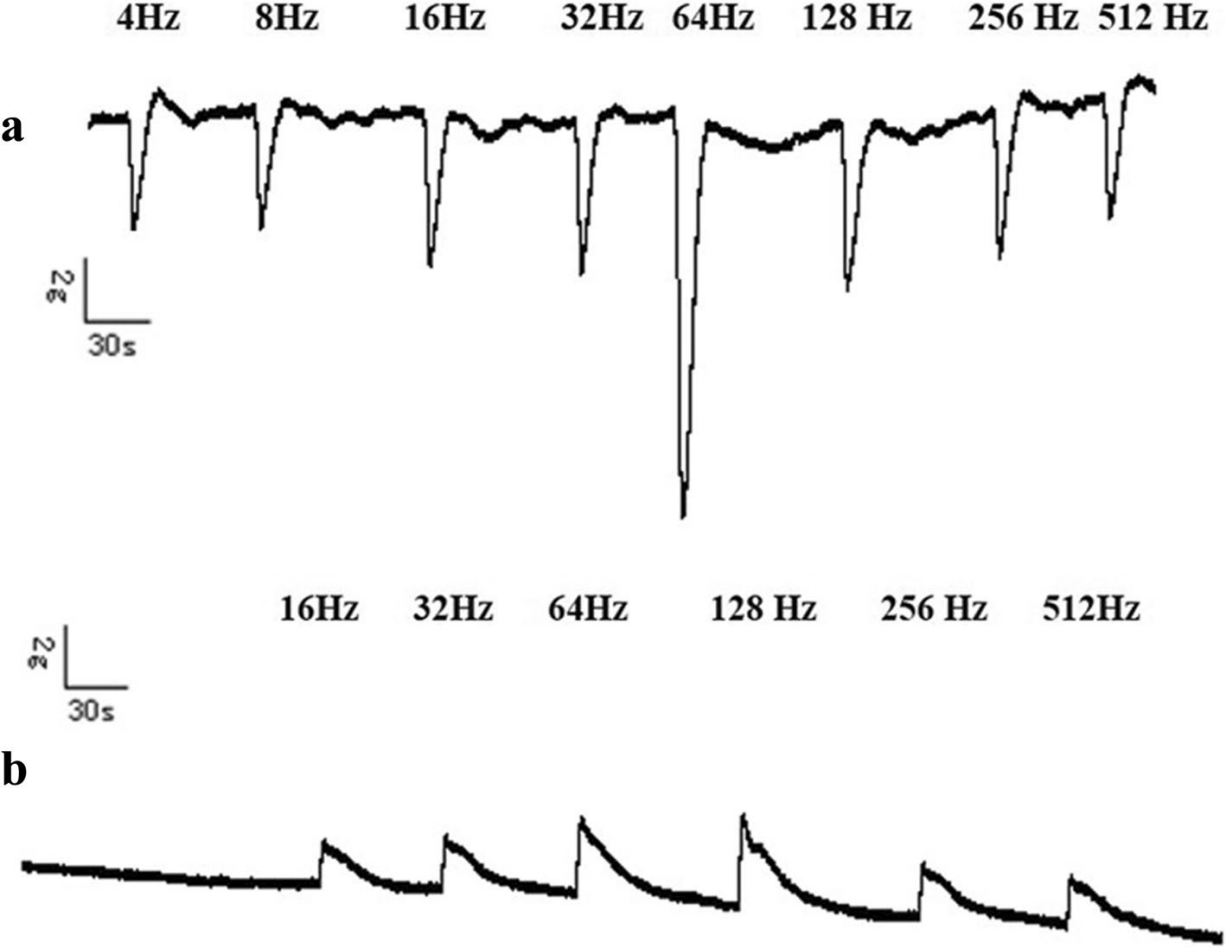
Effect of EFS on the clasp and sling fibers. **a** The EFS induced a frequency-dependent relaxation in clasp fiber. After each of the stimulation, there was a quick rebound contraction which was also in the frequency-dependent manner. The optimal frequency resulting in maximum relaxation was 64 Hz. **b** EFS induced frequency-dependent contraction in the sling fiber, the optimal frequency leading to maximum contraction was 128 Hz. EFS: Electrical field stimulation

EFS could induce frequency dependent relaxation of clasp fiber muscle strips. The electrical stimulation frequency at the maximum relaxation was 64 Hz, When the relaxation response caused by each stimulation was over, the muscle strips immediately appeared rebound contraction, which also showed a frequency-dependent mode. The maximum relaxation percentage of clasp fiber muscle strips induced by EFS was (17.5 ± 1.7) %. The response of sling fiber muscle strips to EFS was frequency dependent contraction, and the electrical stimulation frequency at the maximum contraction was 128 Hz. The maximum contraction percentage of sling fiber muscle strips induced by EFS was (13.8 ± 1.5) %. (Fig. 2a, b) (Tables 1 and 2).

**Table 1:** The relaxation induced by the EFS in the clasp fiber

Groups	Percent relaxation (%)				
	8 Hz	16 Hz	32 Hz	64 Hz	128 Hz
Clasp fiber	2.9 ± 0.2	4.4 ± 0.6	12.5 ± 1.1	17.5 ± 1.7	11.9 ± 1.2

**Table 2:** The contraction induced by the EFS in the sling fiber

Groups	Percent contraction (%)				
	32 Hz	64 Hz	128 Hz	256 Hz	512 Hz
Sling fiber	6.2 ± 0.5	11.6 ± 0.8	13.8 ± 1.5	11.8 ± 1.1	10.3 ± 0.7

### The role of selective LPA1 and LPA3 receptor antagonist Ki16425 in EFS-induced frequency-dependent relaxation of clasp fiber

**Fig. 3:**
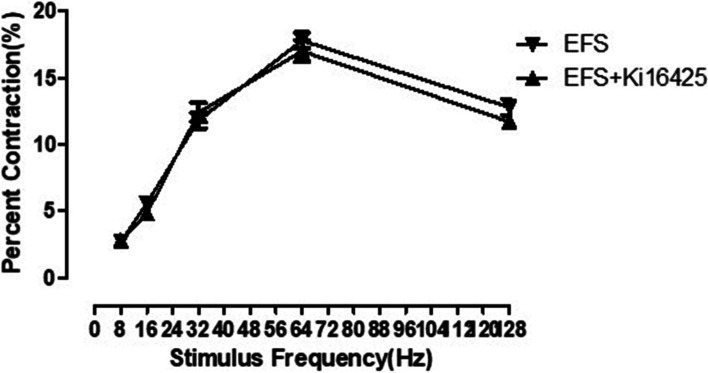
Effect of the selective LPA1 and LPA3 receptor antagonist (Ki16425) on the EFS relaxation of the clasp fiber. The selective LPA1 and LPA3 receptor antagonist produced no significant change in the responses (*P* > 0.05). LPA: Lysophosphatidic acid; EFS: Electrical field stimulation

After EFS induced frequency-dependent relaxation of clasp fiber muscle strip, the selective LPA1 and LPA3 receptor antagonist ki16425 (10^−5^ mol / L) didn’t affect the relaxation of clasp fiber muscle strip, there was no significant difference in relaxation effect before and after adding drugs (*F* = 0.37, *P* = 0.85) (Fig. 3) (Table 3).

**Table 3:** The relaxation of the clasp fiber induced by EFS and EFS + ki16425

Groups	Percent relaxation (%)				
	8 Hz	16 Hz	32 Hz	64 Hz	128 Hz
EFS	2.8 ± 0.2	4.3 ± 0.7	12.6 ± 0.9	18.0 ± 1.6	12.0 ± 0.9
EFS + ki16425	3.1 ± 0.4	4.7 ± 0.6	11.9 ± 1.2	17.4 ± 1.8	11.7 ± 1.2

### The role of selective LPA1 and LPA3 receptor antagonist Ki16425 in EFS-induced frequency-dependent contraction of sling fiber

**Fig. 4:**
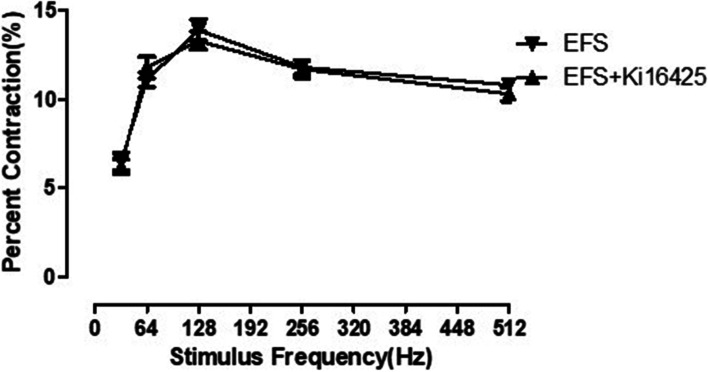
Effect of the selective LPA1 and LPA3 receptor antagonist (Ki16425) on the EFS contraction of the sling fiber muscle strip. The selective LPA1 and LPA3 receptor antagonist produced no significant change in the responses (*P* > 0.05). LPA: Lysophosphatidic acid; EFS: Electrical field stimulation

After EFS induced frequency-dependent contraction of sling fiber muscle strip, the selective LPA1 and LPA3 receptor antagonist ki16425 (10^−5^ mol / L) didn’t affect the relaxation of sling fiber muscle strip, there was no significant difference in contraction effect before and after adding drugs (*F* = 0.67, *P* = 0.63) (Fig. 4) (Table 4).

**Table 4:** The contraction of the sling fiber induced by EFS and EFS + ki16425

Groups	Percent contraction (%)				
	32 Hz	64 Hz	128 Hz	256 Hz	512 Hz
EFS	6.4 ± 0.4	11.2 ± 1.1	14.6 ± 1.7	11.8 ± 0.9	10.6 ± 0.8
EFS + ki16425	5.9 ± 0.5	11.6 ± 0.6	13.6 ± 1.1	11.5 ± 1.4	10.5 ± 0.6

## Discussion

The regulation mechanism of LES contraction and relaxation is controlled by the central nervous system, and is completed by a variety of hormones, neurotransmitters and their own myogenic factors. The output nerve endings of vagus nerve form synapses with EMN located in LES muscle layer [12, 13], which constitutes the neural signal pathway regulated by LES. These EMN can be divided into excitatory motor neurons and inhibitory motor neurons according to their functions. Inhibitory intestinal motor neurons can release NO and cause LES relaxation [14], and excitable intestinal motor neurons can release acetylcholine (Ach) to cause LES contraction [15]. These two kinds of neurons exist simultaneously in LES muscle layer [16], Various hormones, neurotransmitters and their own myogenic factors act on the intrinsic neurons in the muscle layer of LES to form different neural signal transduction pathways, thus completing the regulation of esophageal body contraction, LES contraction and relaxation, and LES resting pressure and other functions [17]. EFS has been widely used in the study of regulation mechanism of LES due to its direct activation of EMN [18].

Previous studies on mouse LES found that EFS could produce frequency dependent relaxation reaction, and rebound contraction occurred after relaxation reaction. It is found that this frequency-dependent relaxation and rebound contraction can be inhibited by nitric oxide synthase (NOS) inhibitor Nω-nitro-L-arginine methyl ester (L-NAME), which fully indicates that NO is involved in the relaxation and rebound contraction of mouse LES induced by EFS [19]. Whether other neurotransmitters are involved in the completion of relaxation response is unclear.

Our previous results confirmed that there are three smooth muscle LPA receptors (LPA1 receptor, LPA2 receptor, LPA3 receptor) in human LES, and their selective agonists induced concentration-dependent contraction in human LES, suggesting that these three receptors may be involved in the regulation of contraction in human LES [7]. Whether the three LPA receptors play a role in EFS-induced reaction of human LES needs further study.

In this study, we found that EFS could induce frequency-dependent relaxation of uncinate fibers. The maximum relaxation frequency was 64 Hz, rebound contraction occurs in muscle strips after relaxation caused by each stimulation. The maximal relaxation percentage of clasp fiber induced by EFS was (17.5 ± 1.7) %. On the other hand, the response of sling fiber to EFS is frequency-dependent contraction, and the frequency of electrical stimulation during maximum contraction is 128 Hz. The maximum percentage of contraction induced by EFS was (13.8 ± 1.5) %.

Our previous studies found that 10^−5^ mol/L selective LPA1, LPA2 receptor agonist and selective LPA3 receptor agonist can induce the maximum contraction effect of clasp and sling fiber muscle strips [20]. Combined with the above research results, in this study, we compared the response of human LES induced by EFS, before and after the application of 10^−5^ mol / L selective LPA1 and LPA3 receptor antagonists, in order to clarify the role of LPA1 and LPA3 receptors in EFS. It was found that there was no significant difference in frequency-dependent relaxation response induced by EFS before and after the use drug in clasp fiber muscle strip. In sling fiber muscle strip, there was no significant difference in frequency-dependent contraction response induced by EFS before and after use drug. It is suggested that LPA receptor is not involved in the response of LES induced by EFS.

It is generally believed that the output nerve endings of vagus nerve and the EMN in the muscle layer of LES constitute the excitatory vagus nerve pathway and the inhibitory vagus nerve pathway. The regulation of LES is accomplished through the two neural pathways. The excitatory vagus nerve pathway comprises preganglionic cholinergic neurons and postganglionic cholinergic neurons. The inhibitory vagus nerve pathway comprises preganglionic cholinergic neurons and NANC neurons [5]. It was found that the excitatory pathway can release Ach and Substance P(SP), and the release of these two neurotransmitters can cause LES contraction [9, 21]. In the inhibitory neural pathway, NANC neurons can release neurotransmitters such as NO, Adenosine triphosphate (ATP) and VIP, and the release of these neurotransmitters can cause relaxation of LES [22, 23]. Relevant experiments have not confirmed whether LPA is included in these neurotransmitters.

Our results suggest that LPA receptor is not involved in the response of LES induced by EFS, which suggests that LPA is not a neurotransmitter released by EMN and is not involved in the regulation of LES. However, our studies have confirmed that the LPA receptors play an important role in the regulation of human LES [20]. However, based on the current research results, the affinity index pD2 of LPA receptor and its agonist and the antagonistic parameter pA2 of its antagonist cannot be accurately calculated. Therefore, the concentration of selective LPA1 and LPA3 receptor antagonists used in this study cannot successfully antagonize LPA receptor-mediated contraction and relaxation induced by EFS. These are just inferences. The possible signal transduction pathway of the LPA receptor in this process needs further investigation.

The present study has several limitations. First, it is a single-center study, and needs multicenter research to further verify these results. Second, the present study is an in vitro study, and we need to verify the results in the vivo studies. Third, LPA 2 receptor has no specific antagonists, and therefore, this may have an impact on the accuracy of the results. Finally, this is the first report on lysophosphatidic acid receptors' contribution to the response of human LES under the EFS.

In conclusion, we found that the EFS induced a frequency-dependent relaxation in clasp fibers and contraction in sling fibers. The LPA 1 and 3 receptors are not involved in the response of clasp and sling fibers of the human lower esophageal sphincter induced by the EFS. Because there is no selective LPA2 antagonist at present, previous research has confirmed that blocking the LPA1 and LPA3 receptors with selective antagonists didn’t affect, yet using a nonspecific antagonist blocks the response [20]. Therefore, the LPA2 receptors could be responsible for blocking the response. It is necessary to further study the intracellular signal transduction pathway of LPA receptor in human LES to clarify the role of LPA receptor in the regulatory mechanism of human LES. It may play an important role in the future treatment of esophageal motor function diseases.

## Data Availability

The datasets used and/or analysed during the current study are available from the corresponding author on reasonable request.
